# Ingested nitrate and nitrite and end-stage renal disease in licensed pesticide applicators and spouses in the Agricultural Health Study

**DOI:** 10.1038/s41370-023-00625-y

**Published:** 2024-01-08

**Authors:** Dazhe Chen, Christine G. Parks, Laura E. Beane Freeman, Jonathan N. Hofmann, Rashmi Sinha, Jessica M. Madrigal, Mary H. Ward, Dale P. Sandler

**Affiliations:** 1 Epidemiology Branch, National Institute of Environmental Health Sciences, NIH, Research Triangle Park, NC USA; 2 Occupational and Environmental Epidemiology Branch, Division of Cancer Epidemiology and Genetics, National Cancer Institute, NIH, Rockville, MD USA; 3 Metabolic Epidemiology Branch, Division of Cancer Epidemiology and Genetics, National Cancer Institute, NIH, Rockville, MD USA

**Keywords:** Nitrate, Nitrite, Water pollution, Processed meat, End-stage renal disease, Chronic kidney disease

## Abstract

**Background:**

Nitrate and nitrite ingestion has been linked to kidney cancer, possibly via the endogenous formation of carcinogenic *N*-nitroso compounds. These exposures might also contribute to end-stage renal disease (ESRD).

**Objectives:**

We investigated associations of drinking water nitrate and dietary nitrate and nitrite intakes (total and by food type) with incident ESRD in the Agricultural Health Study. We also explored modifying effects of vitamin C and heme iron intake, which may affect endogenous nitrosation.

**Methods:**

We performed complete case analyses among private pesticide applicators and their spouses. We obtained water nitrate estimates for participants whose primary drinking water source at enrollment (1993−1997) was public water supplies (PWS) or private wells (*N* = 59,632). Average nitrate concentrations were computed from historical data for PWS users and predicted from random forest models for private well users. Analysis of dietary nitrate and nitrite was restricted to the 30,177 participants who completed the NCI Dietary History Questionnaire during follow-up (1999−2003). Incident ESRD through 2018 was ascertained through linkage with the U.S. Renal Data System. We estimated adjusted hazard ratios (HRs) and 95%CI for associations of tertiles (T) of exposure with ESRD overall and explored effects in strata of vitamin C and heme iron intake.

**Results:**

We identified 469 incident ESRD cases (206 for dietary analysis). Water nitrate and total dietary nitrate/nitrite were not associated with ESRD, but increased ESRD was associated with nitrate and nitrite from processed meats. We found apparent associations between nitrite and ESRD only among participants with vitamin C <median (T3 vs. T1 HR: 2.26, 95%CI: 1.05, 4.86) and with heme iron ≥median (T3 vs. T1 HR: 1.73, 95%CI: 0.89, 3.39).

**Significance:**

ESRD incidence was associated with dietary nitrate/nitrite from processed meat among all study participants and with total dietary nitrite among participants with lower vitamin C or higher heme iron intake.

**Impact statement:**

There are few well-established environmental risk factors for end-stage renal disease (ESRD), a worldwide public health challenge. Ingestion of nitrate and nitrite, which may lead to endogenous formation of carcinogenic *N*-nitroso compounds, has been linked to some cancers and chronic diseases. We investigated these exposures in relation to ESRD in an agricultural cohort. ESRD incidence was associated with dietary nitrate/nitrite from processed meat and with total dietary nitrite among subgroups with lower vitamin C or higher heme iron intake. This study provides preliminary evidence that points to dietary nitrite and possibly dietary nitrate intake as a potential contributor to ESRD.

## Introduction

End-stage renal disease (ESRD), the final stage of chronic kidney disease (CKD) for which dialysis and transplantation are needed, is a worldwide public health challenge [1]. In 2019, there were an estimated 809,103 patients living with ESRD in the United States (U.S.) [2]. Patients with CKD are at increased risk of morbidity and mortality from cardiovascular disease, infections, and other clinical complications [3, 4]. The risk of mortality rises exponentially with progressively worsening kidney function [5, 6], with patients in the advanced stage having 10−30 times the risk of cardiovascular mortality compared to the general population [3]. In addition, ESRD imposes high costs to patients and society; Medicare-related expenditures for beneficiaries with ESRD reached $51.0 billion in the U.S. in 2019 (ref. 2).

Diabetes and hypertension are the leading contributors to ESRD and CKD in general [7, 8]. However, a substantial number of ESRD cases remain unexplained by diabetes, hypertension, or other established risk factors, especially in developing countries [9]. In the past two decades, an epidemic of CKD unrelated to these traditional causes (i.e., CKD of unknown etiology (“CKDu”)) has emerged in various parts of the world with high agricultural activity, including Mesoamerica and parts of South Asia [10, 11]. The etiology of CKDu is likely multi-factorial, but researchers have suggested environmental/occupational exposures to toxic chemicals and chronic dehydration as important contributors [9].

Recently, a study in California identified nitrate in drinking water as a potential novel risk factor for unexplained ESRD cases [12]. Inorganic nitrate is a common surface and groundwater contaminant in agricultural areas, arising from nitrogen-based fertilizers and animal/human organic wastes [13]. By mapping the groundwater nitrate concentrations in various parts of the state and overlaying them with hot spots of unexplained ESRD, researchers associated 80% of the hot spots with elevated levels of nitrate [12]. High nitrate concentrations in groundwater were also detected in parts of Sri Lanka and Western Australia where there were excess numbers of CKD cases [14, 15]. Besides contaminated drinking water, other major sources of nitrate exposure in humans include consumption of specific vegetables/fruits and processed meats. These foods are also major sources of nitrite intake. Higher consumption of high-nitrate containing vegetables has been linked to elevated odds of CKD [16]. Because nitrosation of nitrite forms carcinogenic *N*-nitroso compounds (NOCs) [17, 18], nitrate (which is converted to nitrite by bacteria in the mouth and digestive tract) and nitrite exposures have been investigated in relation to renal cell carcinoma and other cancers [19, 20]. Several NOCs have been shown to cause genotoxicity in human kidney cells [21]. Studies of animal models have also associated NOCs with increased oxidative stress, markers of glomerular damage, and histological changes in kidneys [22, 23]. However, few studies have evaluated these chemicals in relation to ESRD risk. We evaluated the impact of nitrate from drinking water and nitrate and nitrite from diet on incidence of ESRD in the Agricultural Health Study (AHS), one of the few cohort studies that assessed nitrate levels among private well users using validated models and had available data on dietary nitrate and nitrite intake.

## Methods

### Study population

The AHS is a prospective cohort of 89,655 licensed pesticide applicators and their spouses in North Carolina (NC) and Iowa (IA) [24]. Pesticide applicators in NC were private applicators (i.e., farmers), and applicators in IA included both private and commercial applicators. At enrollment (1993-1997), participants completed a self-administered questionnaire that ascertained information on socio-demographics, lifestyle, health, residential address, year moved into the home, primary drinking water source (spouses only), and pesticide use. In the phase 2 (P2) computer-assisted telephone interview (1999−2003), participants were asked to provide updates on health and to complete the National Cancer Institute (NCI) Diet History Questionnaire (DHQ) [25]. We restricted our analysis to 84,739 private applicators and their spouses. Commercial applicators (*N* = 4916) were excluded because they differed in important ways that could affect the risk of ESRD, including socio-demographics, lifestyle, and agricultural exposure patterns [24].

A flowchart for how we arrived at the analytical samples for the water nitrate and dietary nitrate/nitrite analyses is provided in supplemental materials (Fig. S1). In drinking water nitrate analysis, we began follow-up at study enrollment, thereby excluding 72 ESRD cases diagnosed prior to enrollment. Among the 84,667 participants without prevalent ESRD, we excluded 12,494 for whom we could not determine their primary drinking water source and 3025 whose primary drinking water source was neither a private well nor public (community) water supply (PWS). Among the 69,148 participants whose drinking water source was a private well or PWS, we further excluded 6857 participants with missing water nitrate exposure estimates. Finally, we excluded 2659 participants with missing covariates needed for analysis and reached a final analytical sample of 59,632 participants (70.4% of those eligible for the analysis).

The study population for the dietary nitrate/nitrite analysis included 33,254 participants who completed the DHQ. We began follow-up on date of P2 questionnaire completion, thereby excluding 44 ESRD cases diagnosed prior to completing the questionnaire. Among the 33,210 participants without prevalent ESRD, we excluded 2080 with missing dietary nitrate/nitrite data. Finally, we excluded 953 participants with missing covariates needed for analysis and reached a final analytical sample of 30,177 participants (90.9% of those eligible for the analysis). The institutional review board of the National Institutes of Health oversees the study.

### Exposure assessment

The method for developing water nitrate exposure estimates for participants in the AHS has been described elsewhere [26–28]. At enrollment, spouses of applicators were asked to select their primary residential drinking water source from the following choices: private well, PWS, bottled water, or some other source. Those who reported using private wells were also asked to provide well depth and whether the well was cased (i.e., lined to maintain well structure and protect well water from debris). We assigned private applicators the same drinking water source as that reported by their spouses, assuming that applicators and spouses lived together at enrollment. For those who did not report drinking water source at enrollment but provided such information in subsequent interviews, we assigned the water source using their responses in later interviews (see Manley et al. [26] for details).

For PWS users, we linked participants to the PWS (including rural water supplies) serving their homes based on their geocoded address at enrollment. Water nitrate data for water systems in IA from 1987 to 2018 were obtained from the Center for Health Effects of Environmental Contamination at the University of Iowa, and data for NC from 1977 to 2018 were obtained from the North Carolina Department of Environmental Quality. Most PWS data were linked to towns by town names, and remaining PWS were linked via other approaches (e.g., internet searches, phone calls to utilities) [26]. Average exposure to nitrate, measured as nitrate-nitrogen (NO_3_-N, milligram/liter (mg/L)), was computed by averaging annual PWS measurements across years that participants lived in their residence within 1990−2000, a time period around study enrollment. When there were multiple measurements reported in a year, we first computed an annual average and then averaged these annual means over the years of residence. Because duration of residence was ascertained at enrollment among spouses but not among applicators, we assigned applicators years of residence based on residence information assessed in the Phase 3 (2005−2010) interview. For applicators who did not respond to the Phase 3 interview, we assigned them the same years of residence as their spouses, assuming that they lived together. If duration at the enrollment residence was not available (28.1% among PWS users), we averaged annual PWS means across 1990−2000, a time period around study enrollment.

For private well users, random forest classification models were used to predict water nitrate exposures around time of study enrollment. Modeling was performed separately for participants in IA [28] and in NC [27]. Models were trained and tested using private well nitrate measurements (not specific to AHS participants) and nitrate predictors compiled from various sources. Nitrate measurements used in the IA models included 34,084 groundwater NO_3_-N measurements compiled from several water monitoring programs in 1980−2011. Nitrate measurements used in the NC models consisted of 22,059 groundwater NO_3_-N measurements collected by the North Carolina Department of Health and Human Services in 1990−2011. Final models included parameters that were significant predictors of nitrate levels in water, including well depth and location, nearby land use and nitrogen inputs (e.g., animal waste, fertilizers, and septic systems), population density, soil characteristics, and other geologic and meteorological factors. We used multiple years of data whenever they were available to account for potential temporal changes in nitrate predictors (e.g., land use) and to increase predictive performance of models. Using nitrogen predictor variables specific to time of enrollment, we ran state-specific random forest models to predict water nitrate exposures for a period of time around study enrollment. AHS participants’ geocoded enrollment addresses were assumed to approximate the locations of their private wells and were used to derive the predictive model variables to estimate their well water nitrate concentrations. For 26.5% of participants in IA and 23.0% of participants in NC who were missing well depth, an important predictor in the random forest models, the median well depth for the state was used in the models for nitrate prediction.

Nitrate (mg NO_3_/day) and nitrite (mg NO_2_/day) from foods were estimated using a 144-item DHQ developed by the NCI using methods previously described [29]. Participants reported frequency of consumption and portion size for each food consumed over the past 12 months. Nitrate and nitrite contents of foods were estimated after a literature review of 26 studies and reports published between 1967 and 2008 primarily on food products in the U.S. and Canada. Daily intakes of nitrate and nitrite were calculated by multiplying the frequency of consumption of each food by the portion size and the nitrate and nitrite content of each food and summing across all food items. We estimated nitrate and nitrite intake overall, and separately for the following sources of intake: plant, animal, and processed meat (turkey or chicken cold cuts, luncheon or deli-style ham, other cold cuts or luncheon meats, hot dogs or frankfurters, baked ham or ham steak, liverwurst, bacon, sausage). To estimate the total intake of vitamin C, a potential inhibitor of endogenous nitrosation, the frequency of consumption of vitamin C-containing foods and dietary supplements was multiplied by the portion size and the vitamin C levels [30]. Similarly, total heme iron intake, a potential enhancer of nitrosation, was estimated by multiplying the frequency of consumption of heme iron-containing foods by the portion size and the heme content in each food [31].

### Outcome assessment

Incident ESRD diagnosed between study enrollment and June 30, 2018 was ascertained through linkage with the U.S. Renal Data System (USRDS). The USRDS collects data on all ESRD cases in the U.S. through Medical Evidence Form CMS-2728, which is required for all new patients with ESRD, regardless of Medicare eligibility. The USRDS derives the first ESRD service date by taking the earliest of: [1] the date of the start of dialysis for chronic renal failure, as reported on the Medical Evidence form, [2] the date of a kidney transplant, or [3] the date of the first Medicare dialysis claim. The first service date was used to estimate age at the ESRD diagnosis. Cases occurring after study enrollment were counted as incident ESRD. Linkage to the state mortality files and the National Death Index was used to ascertain vital status of the participants until June 30, 2018. The USRDS also captures data on those who die of renal failure when a renal provider submits a required ESRD Death Notification (CMS-2746) form [32]. For analysis of water nitrate exposure, we followed participants from age at enrollment through the first of ESRD event, death from other causes, withdrawal from the study, or end of follow-up (June 30, 2018). For analysis of dietary nitrate/nitrite exposures, we followed participants from age at P2 questionnaire completion.

### Covariates

We identified potential confounders for adjustment based on covariates’ associations with the exposures and the outcome as shown in published literature and availability of data. All covariates were self-reported or derived from information that was self-reported at enrollment and included the following: age (in years), sex (male; female), race (White; Black; American Indian or Alaskan Native; Asian or Pacific Islander; other race), Hispanic ethnicity, highest educational attainment (≤high school; some college or vocational degree; college or more), state (IA; NC), cigarette smoking (never; former; current), body mass index (BMI; in kg/m²: underweight or normal [<25]; overweight [25- < 30]; obese [≥30]), diagnosis of diabetes before enrollment, and lifetime days use of any pesticides (derived from duration and frequency of pesticide use).

### Statistical modeling

We used Cox proportional hazards models to assess the associations of incident ESRD event with drinking water nitrate exposure and dietary nitrate and nitrite exposures, with age as the timescale. In analysis of water nitrate exposure, we calculated hazard ratios (HRs) adjusted for sex, education, state, and smoking. In analyses of dietary exposures, we examined ESRD in relation to dietary nitrate and nitrite intakes total and from plant, animal, and processed meat, respectively, in models additionally adjusted for daily total energy intake ascertained from the DHQ. In addition to fully-adjusted models, we also examined hazards of ESRD in minimally adjusted models that only accounted for age at enrollment to explore the magnitude of confounding. In all models, we used tertile of exposure levels for analysis, with participants in the lowest tertile (T1) of exposure as the referent group. We did not adjust for race or ethnicity in these analyses, despite them being potential confounders of the associations, because of the small number of participants who were non-White or Hispanic.

In sub-analyses of water nitrate and total dietary nitrate/nitrite, we examined effect measure modification (EMM) of the associations by above/below median intakes of vitamin C (146.3 mg/day) and heme iron (403.8 mg/day), which have been shown to inhibit and enhance endogenous nitrosation, respectively [33, 34]. In the U.S., the age- and race/ethnicity-adjusted incidence of ESRD among men was 1.6 times that among women [2]. To explore potential EMM of the associations by sex, we conducted subgroup analyses among male pesticide applicators and among female spouses because the vast majority of pesticide applicators were men and almost all the spouses were women. We also explored EMM of the associations by state (IA vs. NC) to account for any differences (e.g., lifestyles, occupational and residential exposures) in populations [26] that were not captured by existing covariates. In analysis of water nitrate, we stratified by drinking water source (private well vs. PWS) to account for other potential contaminants in private wells and PWS (e.g., pesticides) between participants using these drinking water sources that could have led to adverse renal effects. In addition to assessing EMM in stratum-specific analyses, we also formally tested heterogeneity of the associations by modifiers by including a product term between tertile exposure and the modifier in the model and reported the *p*-value for the joint Wald test.

In water and dietary exposure analyses, we examined the impact of pre-enrollment diabetes by excluding the small group of people with these diagnoses. In another sensitivity analysis, we additionally adjusted for BMI, which has been associated with ESRD risk in many studies [4]. We did not adjust for this covariate in the main analyses because BMI may act as a mediator of the associations between nitrate/nitrite exposures and ESRD, as studies have shown nitrate/nitrite intakes could help regulate energy and lipid metabolism via the nitrate/nitrite/NO pathway [35], thus influencing participants’ BMI. Because pesticide exposure may also be related to ESRD risk [36, 37], we accounted for this co-pollutant exposure by adjusting for lifetime days of use of any pesticides in analyses to see if results would differ. We also explored the potential for cohort effect on the analyses by additionally adjusting for year of enrollment. In both water nitrate and dietary analyses, we assessed the impact of extreme exposure on ESRD risk by further classifying participants in T3 of exposure by the 90th percentile of exposure and compared risk with participants in T1 of exposure.

In analysis of water nitrate exposure, we explored alternative exposure-response trends by examining exposure categorized in quartiles. To explore the effect of cumulative exposure to nitrate from drinking water, we conducted an analysis restricting to participants who resided at their enrollment address for at least 10 years. In the dietary analysis, we adjusted for calorie intake using an alternative method that calculated nutrient densities for nitrate and nitrite (i.e., mg per day per 1000 kilocalories) [38], which were used to categorize participants into tertiles for analysis.

Analyses were conducted using AHS data files releases: P1REL201701.00, P2REL201701.00, and AHSREL202201.00. All analyses were performed using SAS, version 9.4 (SAS Institute Inc., Cary, NC, U.S.). An alpha level of 0.05 was considered statistically significant for all analyses.

## Results

Characteristics of analytical samples for the water nitrate and dietary nitrate/nitrite analyses differed by ESRD status (Table 1). ESRD cases were more likely to be older, male, former smoker, to reside in NC, to receive high school education or less, and to have a pre-enrollment diabetes diagnosis. Cases were also more likely to be obese and to have lived in the enrollment address for longer time, although these covariates had more missing.

**Table 1:** Characteristics of analytical samples for analyses of water nitrate and dietary nitrate/nitrite, stratified by ESRD status, in the AHS.

Characteristic	Water nitrate analysis^a^		Dietary analysis^b^	
	ESRD case	Non-case	ESRD case	Non-case
	*(n* = *469)*	*(n* = *59,163)*	*(n* = *206)*	*(n* = *29,971)*
	*n (%)*	*n (%)*	*n (%)*	*n (%)*
Age at enrollment (years)				
<40	40 (8.5)	17,479 (29.5)	14 (6.8)	7596 (25.3)
40−49	84 (17.9)	17,211 (29.1)	33 (16.0)	8633 (28.8)
50−59	170 (36.3)	13,923 (23.5)	82 (39.8)	7744 (25.8)
≥60	175 (37.3)	10,550 (17.8)	77 (37.4)	5998 (20.0)
Participant				
Pesticide applicator	328 (69.9)	33,792 (57.1)	130 (63.1)	16,044 (53.5)
Spouse	141 (30.1)	25,371 (42.9)	76 (36.9)	13,927 (46.5)
Sex				
Male	327 (69.7)	33,179 (56.1)	129 (62.6)	15,663 (52.3)
Female	142 (30.3)	25,984 (43.9)	77 (37.4)	14,308 (47.7)
State of residence				
Iowa	237 (50.5)	40,363 (68.2)	122 (59.2)	22,390 (74.7)
North Carolina	232 (49.5)	18,800 (31.8)	84 (40.8)	7581 (25.3)
Race				
non-White	37 (7.9)	1042 (1.8)	6 (2.9)	314 (1.1)
White	432 (92.1)	58,067 (98.2)	200 (97.1)	29637 (99.0)
Missing	0	54	0	20
Hispanic ethnicity				
No	437 (98.9)	56,676 (99.0)	194 (98.5)	28,990 (99.2)
Yes	5 (1.1)	550 (1.0)	3 (1.5)	235 (0.8)
Missing	27	1937	9	746
Educational attainment				
≤High school diploma/GED	316 (67.4)	29,817 (50.4)	128 (62.1)	14,334 (47.8)
Some college/2-year degree	93 (19.8)	17,798 (30.1)	45 (21.8)	9239 (30.8)
College graduate	60 (12.8)	11,548 (19.5)	33 (16.0)	6398 (21.4)
Smoking status				
Never smoked	232 (49.5)	36,556 (61.8)	107 (51.9)	19,103 (63.7)
Former smoker	178 (38.0)	15,145 (25.6)	76 (36.9)	7763 (25.9)
Current smoker	59 (12.6)	7462 (12.6)	23 (11.2)	3105 (10.4)
Reported pre-enrollment diabetes diagnosis				
No	307 (69.6)	55,470 (97.1)	147 (74.2)	28,369 (97.2)
Yes	134 (30.4)	1650 (2.9)	51 (25.8)	804 (2.8)
Missing	28	2043	8	798
Reported pre-enrollment kidney disease (excluding kidney stones) diagnosis				
No	397 (90.2)	56,284 (98.4)	184 (92.9)	28713 (98.2)
Yes	43 (9.8)	946 (1.7)	14 (7.1)	520 (1.8)
Missing	29	1933	8	738
Weight classification				
Underweight or normal (BMI < 25)	89 (21.0)	19,176 (35.6)	47 (23.0)	10,834 (36.5)
Overweight (25 ≤ BMI < 30)	187 (44.2)	23,125 (43.0)	83 (40.7)	12,655 (42.7)
Obese (30 ≤ BMI < 35)	147 (34.8)	11,497 (21.4)	74 (36.3)	6179 (20.8)
Missing	46	5365	2	303
Lifetime days any pesticide use				
Never	84 (19.5)	11,066 (20.5)	35 (18.8)	5549 (20.4)
Low (3-64 days)	100 (23.2)	14,660 (27.2)	43 (23.1)	7545 (27.8)
Medium (88-245 days)	117 (27.2)	14,905 (27.6)	48 (25.8)	7666 (28.2)
High (368-7000 days)	130 (30.2)	13,332 (24.7)	60 (32.3)	6421 (23.6)
Missing	38	5200	20	2790
Duration of residence at the enrollment address				
<10	59 (15.0)	14,170 (28.0)	25 (13.8)	6801 (25.8)
10−19	67 (17.1)	13,841 (27.4)	31 (17.1)	6944 (26.4)
20−29	117 (29.8)	11,503 (22.7)	60 (33.2)	6289 (23.9)
≥30	150 (38.2)	11,070 (21.9)	65 (35.9)	6288 (23.9)
Missing	76	8579	25	3649

*ESRD* end-stage renal disease, *AHS* *Agricultural Health Study,* *GED* General Equivalency Diploma, *BMI* body mass index

^a^Analytical sample for water nitrate analysis. Eligible participants had complete water nitrate estimates and did not have ESRD before study enrollment (1993−1997).

^b^Analytical sample for dietary nitrate/nitrite analysis. Eligible participants had complete dietary nitrate and nitrite intake estimates ascertained from a diet history questionnaire administered (1999−2003) and did not have ESRD before administration of the diet questionnaire.

During a median follow-up of 268 months (range: 1−294 months), 469 out of 59,632 participants examined in the water nitrate exposure analysis developed ESRD. In analysis of dietary nitrate/nitrite intake, 206 out of 30,177 participants had an incident ESRD diagnosis. For the vast majority of participants, the average water nitrate exposure was below the maximum contaminant level (10 mg/L) for nitrate established by the U.S. Environmental Protection Agency (Table S1). Most dietary nitrate and nitrite intake came from plants.

We did not observe an association between tertiles of average water nitrate exposure and hazards of ESRD in either minimally-adjusted or fully-adjusted models (Table 2). In analyses stratified by drinking water source, state, and sex, we did not find evidence of heterogeneity in associations by these potential effect measure modifiers (Table S2). No apparent associations were found in any of the subgroups. When we stratified water nitrate analysis by vitamin C and heme iron intakes, we did not find evidence of EMM by these potential dietary modifiers (Table S3). Because dietary data were available for only 45.4% of the analytical sample for the water nitrate analysis, we also performed an analysis of water nitrate and ESRD among the subset of 27,055 participants with complete dietary data to explore the potential for selection bias (Table S3). Compared to the main analysis, we observed a more elevated but non-statistically significant HR in T2 of exposure in this subgroup analysis. Compared to the analytical sample for the water nitrate analysis, participants in this analysis were more likely to be older, female and Iowa residents (Table S4). We observed similar results in sensitivity analyses in which we, separately, excluded participants with pre-enrollment diabetes and additionally adjusted for BMI, lifetime days use of any pesticides, or year of enrollment (Table S5). No association was apparent when we compared ESRD risk among participants with exposure above the 90th percentile to those in T1 of exposure, examined exposures in quartiles, or restricted to participants who lived on their address at enrollment for at least 10 years (Table S5).

**Table 2:** Associations between average water nitrate exposure and risk of end-stage renal disease among AHS participants (*N* = 59,632), 1993−2018.

Exposure		Total Cases (*n* = 469)	Total N (*n* = 59,632)	Minimally adjusted^a^	Fully adjusted^b^
				HR (95% CI)	HR (95% CI)
Average NO_3_-N (mg/L)	T1 (0−1)	148	19858	Referent	Referent
	T2 (1−2.4)	186	19856	1.18 (0.95, 1.46)	1.02 (0.82, 1.27)
	T3 (2.4−46)	135	19918	0.89 (0.70, 1.12)	0.90 (0.72, 1.14)

*AHS* Agricultural Health Study, *HR* hazard ratio, *CI* confidence interval

^a^Minimally adjusted Cox proportional hazards model accounted for age.

^b^Fully adjusted Cox proportional hazards model accounted for age, sex, state of residence, education, and smoking status.

In fully-adjusted models of dietary exposures, we did not find evidence of positive association between total diet nitrate intake and hazards of ESRD (Table 3). We observed modestly elevated hazards of ESRD among participants in T3 of total dietary nitrite exposure compared to the referent group (HR = 1.36, 95%CI: 0.83, 2.21). When examining dietary nitrate by the food source, nitrate and nitrite intakes from plants were not associated with increased hazards of ESRD. Participants in T3 of exposure to nitrate and nitrite from animal source had non-significantly elevated hazards of ESRD compared to the referent group. We observed monotonic exposure-response trends associating nitrate and nitrite from processed meat with higher hazards of ESRD. In these analyses, statistically significant increases in ESRD hazards were observed in T3 of nitrate exposure (HR = 2.05, 95%CI: 1.37, 3.05) and in T2 (HR = 1.60, 95%CI: 1.09, 2.34) and T3 (HR = 2.22, 95%CI: 1.47, 3.34) of nitrite exposure. Associations in minimally-adjusted models were somewhat stronger, but interpretations remained similar.

**Table 3:** Associations between dietary nitrate and nitrite intake (total and by food source) and risk of end-stage renal disease among AHS participants (N = 30,177), 1999−2018.

Exposure		Total Cases (n = 206)	Total N (n = 30,177)	Minimally adjusted^a^	Fully adjusted^b^
				HR (95% CI)	HR (95% CI)
Nitrate, diet total(mg NO_3_/day)	T1 (4.6−45.1)	62	10052	Referent	Referent
	T2 (45.1−74.5)	54	10047	0.76 (0.53, 1.10)	0.73 (0.50, 1.06)
	T3 (74.5−553.6)	90	10078	1.18 (0.85, 1.64)	1.04 (0.71, 1.52)
Nitrite, diet total(mg NO_2_/day)	T1 (0.1−0.9)	63	10051	Referent	Referent
	T2 (0.9−1.3)	54	10122	0.86 (0.60, 1.24)	0.84 (0.57, 1.24)
	T3 (1.4−4.6)	89	10004	1.52 (1.10, 2.09)	1.36 (0.83, 2.21)
Nitrate, plant source(mg NO_3_/day)	T1 (2.7−39.9)	63	10050	Referent	Referent
	T2 (39.9−68.4)	54	10049	0.72 (0.50, 1.04)	0.69 (0.48, 1.01)
	T3 (68.4−541.3)	89	10078	1.11 (0.80, 1.53)	0.97 (0.67, 1.41)
Nitrite, plant source(mg NO_2_/day)	T1 (0.1−0.5)	61	10077	Referent	Referent
	T2 (0.5−0.8)	56	10254	0.86 (0.60, 1.24)	0.78 (0.53, 1.15)
	T3 (0.8-3.3)	89	9846	1.36 (0.98, 1.88)	1.05 (0.68, 1.62)
Nitrate, animal source(mg NO_3_/day)^c^	T1 (0.1−4)	70	10082	Referent	Referent
	T2 (4−6.3)	61	10019	1.00 (0.71, 1.41)	0.98 (0.68, 1.41)
	T3 (6.3−35.2)	75	10076	1.42 (1.03, 1.98)	1.29 (0.83, 2.00)
Nitrite, animal source(mg NO_2_/day)^c^	T1 (0−0.3)	68	10513	Referent	Referent
	T2 (0.3−0.6)	60	9933	1.02 (0.72, 1.44)	0.99 (0.69, 1.44)
	T3 (0.6−3.2)	78	9731	1.58 (1.14, 2.19)	1.50 (0.97, 2.33)
Nitrate, processed meat(mg NO_3_/day)	T1 (0−0.6)	48	10140	Referent	Referent
	T2 (0.6−1.3)	63	9984	1.42 (0.98, 2.07)	1.35 (0.92, 1.99)
	T3 (1.4−25)	95	10053	2.32 (1.64, 3.28)	2.05 (1.37, 3.05)
Nitrite, processed meat(mg NO_2_/day)	T1 (0−0.1)	46	10237	Referent	Referent
	T2 (0.1−0.2)	70	10234	1.66 (1.14, 2.41)	1.60 (1.09, 2.34)
	T3 (0.2−2.7)	90	9706	2.40 (1.68, 3.43)	2.22 (1.47, 3.34)

*AHS* Agricultural Health Study, *HR* hazard ratio, *CI* confidence interval

^a^Minimally adjusted Cox proportional hazards models accounted for age.

^b^Fully adjusted Cox proportional hazards models accounted for age, sex, state of residence, education, smoking status, and total caloric intake.

^c^Animal source includes processed meat.

In analyses stratified by vitamin C intake, we observed a statistically significant association in T3 of total dietary nitrite intake in the subgroup with vitamin C intake below the median (HR = 2.26, 95%CI: 1.05, 4.86) (Table 4). No association was found between dietary nitrite and ESRD hazards in the subgroup with vitamin C intake above the median, nor for dietary nitrate in either vitamin C subgroup. In analyses stratified by heme iron intake, we observed elevated hazards of ESRD in T3 of dietary nitrite exposure (HR = 1.73, 95%CI: 0.89, 3.39) among participants with heme iron intake above the median (Table 4). In this subgroup, we also observed modestly elevated hazards of ESRD in T3 of dietary nitrate intake (HR = 1.22, 95%CI: 0.72, 2.06). We did not observe positive associations with nitrate or nitrite in the subgroup with heme iron intake below the median. We did not find statistically significant interaction of nitrate and nitrite with vitamin C (interaction-p for nitrate: 0.74, interaction-p for nitrite: 0.69) or with heme iron (interaction-p for nitrate: 0.59, interaction-p for nitrite: 0.70) based on the joint Wald test.

**Table 4:** Associations between dietary nitrate and nitrite exposure and risk of end-stage renal disease among AHS participants, stratified by median vitamin C intake (146.3 mg/day) and median heme iron intake (403.8 mg/day), respectively, 1999−2018.

Exposure	Vitamin C Intake below Median				Vitamin C Intake above Median				Interaction *p*
	Tertile	Total Cases (*n* = 90)	Total N (*n* = 15,086)	HR (95% CI)^a^	Tertile	Total Cases (*n* = 116)	Total N (*n* = 15,091)	HR (95% CI)^a^	
Nitrate, diet total (mg NO_3_/day)	T1 (4.6−45.1)	43	7155	Referent	T1 (4.6−45.1)	19	2897	Referent	0.74
	T2 (45.1−74.5)	28	5234	0.73 (0.44, 1.21)	T2 (45.1–74.5)	26	4813	0.72 (0.40, 1.32)	
	T3 (74.5−330.2)	19	2697	0.87 (0.48, 1.58)	T3 (74.5–553.6)	71	7381	1.12 (0.64, 1.96)	
Nitrite, diet total (mg NO_2_/day)	T1 (0.1−0.9)	35	6534	Referent	T1 (0.1−0.9)	28	3517	Referent	0.69
	T2 (0.9−1.3)	26	5023	1.18 (0.66, 2.09)	T2 (0.9–1.3)	28	5099	0.64 (0.37, 1.11)	
	T3 (1.4−4.3)	29	3529	2.26 (1.05, 4.86)	T3 (1.4−4.6)	60	6475	0.93 (0.49, 1.78)	

	Heme Iron Intake below Median				Heme Iron Intake above Median				Interaction *p*
	Tertile	Total Cases (*n* = 92)	Total N (*n* = 15,088)	HR (95% CI)^a^	Tertile	Total Cases (*n* = 114)	Total N (*n* = 15,089)	HR (95% CI)^a^	
Nitrate, diet total (mg NO_3_/day)	T1 (4.6−45.1)	32	5379	Referent	T1 (4.6−45.1)	30	4673	Referent	0.59
	T2 (45.1−74.5)	21	4888	0.56 (0.32, 0.99)	T2 (45.1−74.5)	33	5159	0.90 (0.54, 1.50)	
	T3 (74.5−539.2)	39	4821	0.88 (0.51, 1.53)	T3 (74.5−553.6)	51	5257	1.22 (0.72, 2.06)	
Nitrite, diet total (mg NO_2_/day)	T1 (0.1−0.9)	38	6313	Referent	T1 (0.1-0.9)	25	3738	Referent	0.70
	T2 (0.9−1.3)	22	5126	0.63 (0.36, 1.12)	T2 (0.9–1.3)	32	4996	1.04 (0.60, 1.82)	
	T3 (1.4−4.1)	32	3649	1.01 (0.49, 2.10)	T3 (1.4–4.6)	57	6355	1.73 (0.89, 3.39)	

*AHS* Agricultural Health Study, *HR* hazard ratio, *CI* confidence interval.

^a^Cox proportional hazards models accounted for age, sex, state of residence, education, smoking status, and total caloric intake.

When we stratified analyses by sex, we observed modestly and non-significantly elevated hazards of ESRD in T3 of dietary nitrite exposure in the male applicator subgroup (Table S6). No apparent association was found for nitrite exposure among female spouses, and nitrate was not associated with ESRD hazards in either subgroup. When we restricted to participants in IA, we did not find an association for dietary nitrate or nitrite intake with ESRD hazards (Table S7). Among participants in NC, we observed a statistically significant association in T3 of nitrite exposure; however, confidence intervals were wide in this subgroup due to the smaller number of participants and cases. There were no substantive differences in results from the main analysis when we excluded participants with pre-enrollment diabetes or when we additionally adjusted for BMI, lifetime days use of any pesticides, or year of enrollment in models (Table S8). We found a statistically significant association comparing risk of ESRD among participants with nitrite exposure above the 90^th^ percentile to that among those in T1 of nitrite exposure; no association was found in analysis of dietary nitrate (Table S8). When we accounted for total energy intake by examining nitrate and nitrite as nutrient densities (i.e., per 1000 kilocalories), we did not observe elevated association for nitrate. We observed elevated but non-statistically significant HRs in T2 or T3 of nitrite exposure (Table S9).

## Discussion

In this study, we examined the relationship of drinking water nitrate and dietary nitrate and nitrite intakes with incident ESRD among pesticide applicators and their spouses in the AHS. We observed only a modest increase in hazards of ESRD among applicators in the top tertile of dietary nitrite exposure compared to the referent group. No apparent association was observed for nitrate exposure from water or diet. In dietary analyses stratified by vitamin C and heme iron intake, we observed elevated hazards of ESRD in T3 of nitrite intake among participants with vitamin C intake below the median or with heme iron intake above the median. The magnitudes of the effect in the highest exposure tertile in these subgroup analyses were comparable to the increase in ESRD risk comparing current smokers (relative risk: 1.91) to never smokers, as estimated in a meta-analysis [39].

Consumption of contaminated drinking water has been proposed as a potential contributor to the endemic CKDu observed in various parts of the world with high agricultural activity [10, 11]. Inorganic nitrate is a common surface and groundwater contaminant in agricultural areas, arising from nitrogen-based fertilizers and animal/human organic wastes [13]. High concentrations of nitrate [15] and measures of fertilizer runoff [40] have been detected in groundwater in Sri Lanka, a country with an large number of CKDu cases; however, these studies lacked data to evaluate associations on the individual level. Groundwater nitrate concentration was not associated with serum creatinine in a crude analysis of Sri Lanka CKDu patients [41].

To our knowledge, only one epidemiologic study has been published on the relationship between water nitrate exposure and incidence of CKD/ESRD. In an ecologic study conducted in California, researchers obtained at the zip code-level, average groundwater nitrate concentrations from wells sampled annually by the California Water Board from 2010 to 2014 and unexplained incident ESRD cases in 2015−2017 from the USRDS [12]. Groundwater nitrate levels superimposed over ESRD hot spots showed that 85% of the hot spots had wells in which nitrate levels exceeded the state median value. In contrast, we did not observe an association between water nitrate exposure and ESRD risk among AHS participants. The average concentration of groundwater nitrate among private well users in our study was comparable to that in the California study. Our study improved upon this previous study by leveraging individual-level data (instead of data on the zip code level) and accounting for potential confounders of the association.

While there are few studies of CKD/ESRD, a few studies have examined water nitrate in relation to kidney cancer. No association was found in a crude ecologic analysis of water exposure and annual incidence of renal cell carcinoma in Germany [42]. In a cohort of postmenopausal women, long-term average nitrate levels >0.36 mg/L in PWS were not associated with kidney cancer in 11 years of follow-up [43]; however, an updated analysis of this cohort with longer follow-up (mean: 21 years) linked elevated risk to exposure above the 95th percentile (>5 mg/L) among participants who reported using their drinking water source for >10 years [44]. We did not observe an association in sensitivity analyses in which we examined exposure above the 90th percentile (>7.3 mg/L) or restricted to participants who lived on their address at enrollment for at least 10 years.

No study has examined the relationship between *dietary* nitrate intake and incidence of CKD/ESRD. In an experimental study of adult men with normal kidney function, dietary supplementation of inorganic nitrate (150 mg three times per day) for a week produced no differences in markers of kidney function compared to the placebo-controlled group [45]. In another study, consumption of total nitrate-containing vegetables or high-nitrate containing vegetables (>100 mg nitrate/100 g vegetable) was not associated with odds of CKD after 3 years of follow-up, although a positive association was found between intake of high-nitrate containing vegetables and odds of CKD at baseline [16].

In our study, we did not observe an association between total dietary nitrate or nitrite with hazards of ESRD. When examining intake by food source, nitrate or nitrite from plants was not associated with ESRD hazards. Higher intakes of nitrate and nitrite from processed meat were associated with clear increases in ESRD hazards, and suggestive associations were also found for total nitrite intake and nitrite from animal source. These results are consistent with studies that investigated kidney cancer and nitrate/nitrite exposure by diet source. In an analysis of the NIH-AARP Diet and Health Study participants, nitrite from processed meat and from other animal sources was associated with higher risk of kidney cancer, but no association was found for nitrite from plants or total nitrate intake [46]. In a study of postmenopausal women, higher risk of kidney cancer was found among participants with higher nitrite intake from processed meat but not with total dietary nitrate or nitrite intake [44].

The heterogenous associations observed for water nitrate and nitrate/nitrite from different food sources in our study could be a result of the presence of precursors, enhancers, and inhibitors in these dietary sources that may differentially affect endogenous nitrosation [47], the proposed biological mechanism for the positive associations observed between exposure to nitrate/nitrite and cancers in some studies [18] and the hypothesized mechanism for our study. Specifically, endogenous nitrosation is the process by which ingested *nitrite* reacts with other nitrosation precursors to form NOCs, which have shown carcinogenic properties in animal studies [48–50]. Several NOCs have been shown to cause genotoxicity in human kidney cells [21]. Studies of animal models have associated NOCs with increased oxidative stress, markers of glomerular damage, and histological changes in kidneys [22, 23]. *Nitrate* also contributes to NOC production, as a small percentage (~5%) of ingested nitrate is reduced to nitrite by commensal bacteria present in the mouth and converted to nitrosating agents in the digestive tract [17]. The endogenous conversion of nitrate to nitrite could be affected by other factors that differ among the individuals (e.g., pH level and temperature of the mouth, use of mouthwash) [51–53], which might explain the weaker and less consistent associations observed for nitrate compared to nitrite in our main and subgroup analyses.

Meat and fish contain amines and amides [54], which are precursors of nitrosation, and red meat contains heme iron, a catalyst of nitrosation [33]. It is plausible that these nitrosation precursors and enhancers resulted in the conversion of nitrite to NOCs after ingestion to produce the elevated HR observed in T3 of nitrite from animal source. Even stronger associations were observed for nitrate and nitrite from processed meat. Nitrite added to processed meat may have resulted in additional formation of NOCs during the curing process [55]. Also, certain high-temperature cooking practices (e.g., frying, grilling) commonly used for processed meats may produce higher amounts of NOCs and other toxicants (e.g., heterocyclic amines, polycyclic aromatic hydrocarbons) with adverse renal effects [56–58]. In contrast, no association was observed between nitrate/nitrite from plants and ESRD. The lack of association could be attributed to the presence of Vitamin C and other antioxidants (e.g., flavonoids) in certain fruits and vegetables, which have been shown to inhibit endogenous nitrosation [34, 59].

To explore these possibilities, we performed sub-analyses of exposures and ESRD hazards stratified by heme iron and vitamin C intakes. We did not find any association between water nitrate exposure and ESRD in strata of heme iron or vitamin C. However, in dietary analyses, we found positive associations for dietary nitrite in subgroups with ≥median heme iron intake or with <median vitamin C intake. In a case-control study of water nitrate and kidney cancer among PWS users, exposure to average nitrate levels >5 mg/L was associated with elevated risk of kidney cancer only in the subset of participants with high red meat intake and low vitamin C intake [60]. In studies of nitrate/nitrite exposure and other cancers, the effect of dietary enhancers and inhibitors of nitrosation varied [19, 20], with some studies showing heterogenous associations between nitrate/nitrite and cancer risk by these dietary mediators [61–63] and other studies finding no differences in associations by them [64, 65].

In the dietary analyses stratified by state, we observed a significantly elevated association in T3 of nitrite exposure among NC residents, while no apparent association was observed in IA residents. Differences in agricultural exposures or lifestyles between the states that were not captured by existing covariates in the model may have made participants in NC more susceptible to the effect of dietary nitrite exposures. For instance, the sub-cohorts in NC and IA have major differences in the size of farms, types of crops planted, and farm animal operations [24]. The higher dietary nitrate and nitrite intake among NC residents compared to IA residents also suggest differences in dietary patterns that could influence risk of ESRD. One major strength of the study is the outcome ascertainment via linkage to the USRDS database. The USRDS identifies the vast majority of ESRD cases in the U.S. from providers, regardless of Medicare eligibility [32]. This reduced concerns about loss to follow-up or outcome misclassification in our study. Another strength is the estimation of long-term quantitative water nitrate estimates not only among PWS users but also among private well users less often studied. Because private wells are not regulated in the U.S., nitrate in private wells are not routinely measured despite the typically higher concentrations in private wells compared to PWS. The AHS is one of the few agricultural cohort studies that collected information on drinking water source and assessed nitrate levels among private well users using validated models. A third strength of the study is the use of a validated DHQ in conjunction with well-developed dietary databases to estimate nitrate intake from foods, as well as dietary modifiers of nitrosation (i.e., vitamin C and heme iron intake) [29]. Lastly, the availability of participant-level data on many important covariates allowed us to account for important confounders of the association in main and sensitivity analyses.

One limitation of the study is the potential for selection bias resulting from restricting to participants with non-missing water nitrate estimates for the water nitrate analysis and to DHQ respondents for the dietary analysis. The water nitrate analysis stratified by vitamin C and heme iron was evaluated among the subset of 27,055 participants who had complete dietary data. A comparison of the enrolled population and these analytical samples showed no major differences in characteristics, mitigating concerns over selection bias (Table S1). Another limitation is that most participants in the study were White and non-Hispanic, which limits generalizability of our study findings to more racially/ethnically diverse populations. We were not able to examine associations by racial and ethnic groups to determine if nitrate/nitrite exposures were a contributor to the higher disease burden among Black and Hispanic persons [2]. Our results may be less generalizable to non-agricultural populations, as studies have shown farm workers to be overall healthier but more susceptible to some diseases, possibly from the various agricultural exposures that they had [66, 67].

The water nitrate exposure estimates contained some degree of uncertainty. We used the median well depth for the state in the private well nitrate prediction models for the 26.5% of IA participants and 23.0% of NC participants with missing values for this covariate. A spatial analysis of well depth data in each state showed no evidence of geographic clustering that supports using median well depths for smaller geographic areas for imputation. This imputation may have resulted in non-differential misclassification in the exposure estimates. We used participants’ enrollment addresses for linkage to the PWS and private wells and estimate water nitrate levels. It is possible that some participants might have lived at a different address and had a different drinking water source before enrollment. For PWS users, we used residential duration-specific average exposure estimates that accounted for the number of years participants resided at the address reported at enrollment whenever this information was available. For those who did not report years of residence, we averaged annual water nitrate measurements across 1990−2000.Among participants with available data on duration of residence, 72% of them lived in their address at study enrollment for at least 10 years. We performed a sensitivity analysis restricting to these participants and found similar (i.e., null) associations compared to the main analysis. We do not expect measurement error in water nitrate to substantively bias our results given that the AHS is a residentially stable cohort of couples mostly living on farms. In our study, we estimated water nitrate exposure for a time period around study enrollment. We did not have exposure data for private well users beyond this exposure window, partly due to our continued effort to verify changes in participants’ addresses and drinking water source after enrollment, and therefore, could not assess the impact of continued exposure on our results.

A fourth limitation is that we examined incident ESRD instead of incident CKD. CKD may develop years before disease progresses to ESRD, which prompts the need for dialysis and kidney transplant [4]. Many CKD cases may go unnoticed until they are sufficiently advanced to be symptomatic. Because the timing of disease onset is not known, it is possible that incident ESRD cases could have CKD that predates study enrollment. We were not able to determine if nitrate/nitrite exposures were related to CKD initiation or to the progression to end stage disease. There were too few people with self-reported kidney diseases (that did not involve dialysis) (Table 1) to consider an analysis of disease progression among those with underlying kidney diseases before enrollment.

Lastly, there could be residual confounding from unmeasured or unknown confounders or imperfect measurement of existing covariates in the models. We did not account for all other occupational or environmental exposures, such as other water contaminants that were related to agricultural activities (e.g., pesticides, heavy metals) and heat stress/dehydration that have been associated with ESRD risk in other studies. We saw no substantive differences in associations in sensitivity analyses adjusted for lifetime days use of any pesticides. We used education as a proxy for socioeconomic disparities, acknowledging that this variable might not have fully captured the socioeconomic effects (e.g., healthcare access and quality) that may have influenced risk of ESRD. We adjusted for pre-enrollment diabetes diagnosis in a sensitivity analysis and found similar results to the main analysis, but we are aware that self-reported diabetes may not be accurate, and there is still a potential for residual confounding.

In this study, we identified a positive association between dietary nitrite from processed meat and ESRD hazards. In stratified analyses, we found increases in ESRD hazards from dietary nitrate and nitrite in the subgroup with higher heme iron intake and from dietary nitrite only among participants with lower vitamin C intake. No associations were found for water nitrite or dietary nitrate/nitrite in the overall study population. To our knowledge, our study is the first to evaluate the relationships between quantitative nitrate/nitrite estimates and incidence of ESRD while accounting for potential confounders of the associations. These results provide preliminary evidence that points to higher dietary nitrite intake (and possibly higher dietary nitrate intake) as a potential contributor to ESRD among certain susceptible groups. Additional research is needed in other non-occupational and more racially/ethnically diverse populations to confirm these study findings. Research examining CKD or biomarkers of earlier renal dysfunction as the outcomes may further clarify the potential risks associated with nitrate and nitrite exposures.

### Supplementary information

Supplementary Tables and Figures

## Data Availability

The authors do not have permission to share the outcome data without explicit consent from the USRDS. Other data from the AHS may be requested following procedures outlined on the study website.
