# Supplementary material for: Ingested nitrate and nitrite and end-stage renal disease in licensed pesticide applicators and spouses in the Agricultural Health Study
Source: J Expo Sci Environ Epidemiol. 2024 Jan 8;34(2):322–32. doi: 10.1038/s41370-023-00625-y (PMC11142909; doi:10.1038/s41370-023-00625-y)
Supplement: Supplementary file 1 — Supplementary Tables and Figures [file 41370_2023_625_MOESM1_ESM.docx]

| Table S1. Distribution (range and quartiles) of water nitrate exposure and dietary nitrate and nitrite exposure among AHS participants | | | | | | |
| --- | --- | --- | --- | --- | --- | --- |
|  | **Minimum** | **Q1 cutpoint** | **Mean** | **Median** | **Q3 cutpoint** | **Maximum** |
| **Average water nitrate (mg NO_3_-N/L)** |  |  |  |  |  |  |
| All study participants | 0.0 | 0.8 | 2.9 | 1.5 | 3.1 | 46.0 |
| Private well users | 0.1 | 1.0 | 3.3 | 1.7 | 3.2 | 46.0 |
| Public water system users | 0.0 | 0.2 | 1.7 | 0.7 | 2.6 | 18.0 |
| Iowa participants | 0.0 | 0.8 | 3.5 | 1.5 | 4.3 | 46.0 |
| North Carolina participants | 0.0 | 0.9 | 1.6 | 1.6 | 2.3 | 12.4 |
| **Dietary nitrate (mg NO_3_/day)** |  |  |  |  |  |  |
| Nitrate, diet total | 4.6 | 39.2 | 69.7 | 57.9 | 86.5 | 553.6 |
| Nitrate, plant source | 2.7 | 34.3 | 64.1 | 52.4 | 80.1 | 541.3 |
| Nitrate, animal source | 0.1 | 3.5 | 5.6 | 5.1 | 7.1 | 35.2 |
| Nitrate, processed meat | 0.0 | 0.5 | 1.3 | 0.9 | 1.7 | 25.0 |
| **Dietary nitrite (mg NO_2_/day)** |  |  |  |  |  |  |
| Nitrite, diet total | 0.1 | 0.8 | 1.2 | 1.1 | 1.5 | 4.6 |
| Nitrite, plant source | 0.1 | 0.5 | 0.7 | 0.7 | 0.9 | 3.3 |
| Nitrite, animal source | 0.0 | 0.3 | 0.5 | 0.4 | 0.6 | 3.2 |
| Nitrite, processed meat | 0.0 | 0.1 | 0.2 | 0.1 | 0.2 | 2.7 |
|  |  |  |  |  |  |  |
| Abbreviations: AHS, Agricultural Health Study | | | | | | |

| Table S2. Effect measure modification of the association between average water nitrate exposure and risk of end-stage renal disease by drinking water source, state of residence, and sex, among AHS participants, 1993-2018 | | | | | | |
| --- | --- | --- | --- | --- | --- | --- |
| **Subgroups** | **Average NO_3_-N (mg/L)** | **Total Cases** | **Total N** | **HR (95% CI)^a^** |  | **Interaction p** |
|  |  |  |  |  |  |  |
| Private well users | T1 (0.1-1) | 77 | 11306 | Referent |  | 0.74 |
|  | T2 (1-2.4) | 173 | 17363 | 1.06 (0.79, 1.42) |  |  |
|  | T3 (2.4-46) | 116 | 16194 | 0.92 (0.69, 1.24) |  |  |
|  |  |  |  |  |  |  |
| PWS users | T1 (0-1) | 71 | 8552 | Referent |  |  |
|  | T2 (1-2.4) | 13 | 2493 | 0.84 (0.44, 1.59) |  |  |
|  | T3 (2.4-18) | 19 | 3724 | 0.87 (0.49, 1.56) |  |  |
|  |  |  |  |  |  |  |
|  |  |  |  |  |  |  |
| Iowa Residence | T1 (0-1) | 84 | 14535 | Referent |  | 0.89 |
|  | T2 (1-2.4) | 65 | 10614 | 1.04 (0.75, 1.44) |  |  |
|  | T3 (2.4-46) | 88 | 15451 | 0.95 (0.70, 1.28) |  |  |
|  |  |  |  |  |  |  |
| North Carolina Residence | T1 (0-1) | 64 | 5323 | Referent |  |  |
|  | T2 (1-2.4) | 121 | 9242 | 0.99 (0.73, 1.34) |  |  |
|  | T3 (2.4-12.4) | 47 | 4467 | 0.83 (0.57, 1.22) |  |  |
|  |  |  |  |  |  |  |
|  |  |  |  |  |  |  |
| Male pesticide applicator | T1 (0-1) | 101 | 11184 | Referent |  | 0.74 |
|  | T2 (1-2.4) | 133 | 11128 | 1.05 (0.81, 1.37) |  |  |
|  | T3 (2.4-46) | 89 | 11038 | 0.89 (0.67, 1.18) |  |  |
|  |  |  |  |  |  |  |
| Female spouse | T1 (0-1) | 41 | 8356 | Referent |  |  |
|  | T2 (1-2.4) | 52 | 8359 | 1.09 (0.72, 1.65) |  |  |
|  | T3 (2.4-46) | 44 | 8641 | 1.01 (0.66, 1.55) |  |  |
|  |  |  |  |  |  |  |
|  |  |  |  |  |  |  |
| Abbreviations: AHS, Agricultural Health Study; HR, hazard ratio; CI, confidence interval | | | | | | |
| ^a^Cox proportional hazards models accounted for age, sex, state of residence, education, and smoking status. | | | | | | |

| Table S3. Association between average water nitrate exposure and risk of end-stage renal disease among AHS participants with complete dietary data and stratified by median vitamin C intake (146.3 mg/day) and median heme iron intake (403.8 mg/day) in the dietary analyses population, respectively, 1993-2018 | | | | | | |
| --- | --- | --- | --- | --- | --- | --- |
| **Subgroups** | **Average NO_3_-N (mg/L)** | **Total Cases** | **Total N** | **HR (95% CI)^a^** |  | **Interaction p** |
|  |  |  |  |  |  |  |
| Analysis among participants with complete vitamin C and heme iron intake estimates | T1 (0-1) | 58 | 9286 | Referent |  | N/A |
|  | T2 (1-2.4) | 85 | 9036 | 1.27 (0.90, 1.80) |  |  |
|  | T3 (2.4-46) | 61 | 9618 | 1.01 (0.70, 1.45) |  |  |
|  |  |  |  |  |  |  |
|  |  |  |  |  |  |  |
| Vitamin C Intake below Median | T1 (0-1) | 27 | 4631 | Referent |  | 0.68 |
|  | T2 (1-2.4) | 37 | 4518 | 1.12 (0.68, 1.86) |  |  |
|  | T3 (2.4-46) | 31 | 4847 | 1.09 (0.65, 1.83) |  |  |
|  |  |  |  |  |  |  |
| Vitamin C Intake above Median | T1 (0-1) | 31 | 4655 | Referent |  |  |
|  | T2 (1-2.4) | 48 | 4518 | 1.48 (0.93, 2.35) |  |  |
|  | T3 (2.4-46) | 30 | 4771 | 0.93 (0.56, 1.54) |  |  |
|  |  |  |  |  |  |  |
|  |  |  |  |  |  |  |
| Heme Iron Intake below Median | T1 (0-1) | 27 | 4617 | Referent |  | 0.27 |
|  | T2 (1-2.4) | 32 | 4705 | 0.95 (0.56, 1.60) |  |  |
|  | T3 (2.4-46) | 25 | 4621 | 0.92 (0.53, 1.59) |  |  |
|  |  |  |  |  |  |  |
| Heme Iron Intake above Median | T1 (0-1) | 31 | 4669 | Referent |  |  |
|  | T2 (1-2.4) | 53 | 4331 | 1.64 (1.04, 2.57) |  |  |
|  | T3 (2.4-43) | 36 | 4997 | 1.07 (0.66, 1.73) |  |  |
|  |  |  |  |  |  |  |
|  |  |  |  |  |  |  |
| Abbreviations: AHS, Agricultural Health Study; HR, hazard ratio; CI, confidence interval | | | | | | |
| ^a^Cox proportional hazards models accounted for age, sex, state of residence, education, and smoking status. | | | | | | |

| Table S4. Characteristics of enrolled participants and analytical samples for main and sub-analyses of water nitrate and dietary nitrate/nitrite, respectively, in the AHS | | | | |
| --- | --- | --- | --- | --- |
| **Characteristic** | **Enrollment^a^** | **Water nitrate analysis^b^** | **Dietary analysis^c^** | **Water nitrate sub-analysis^d^** |
|  | *(n=84,739)* | *(n=59,632)* | *(n=30,177)* | *(n=27,055)* |
|  | *n (%)* | *n (%)* | *n (%)* | *n (%)* |
| Age at enrollment (years) |  |  |  |  |
| < 40 | 26595 (31.4) | 17519 (29.4) | 7610 (25.2) | 6546 (24.2) |
| 40-49 | 23312 (27.5) | 17295 (29.0) | 8666 (28.7) | 7794 (28.8) |
| 50-59 | 18853 (22.3) | 14093 (23.6) | 7826 (25.9) | 7184 (26.6) |
| ≥ 60 | 15979 (18.9) | 10725 (18.0) | 6075 (20.1) | 5531 (20.4) |
| Participant |  |  |  |  |
| Pesticide applicator | 52394 (61.8) | 34120 (57.2) | 16174 (53.6) | 14378 (53.1) |
| Spouse | 32345 (38.2) | 25512 (42.8) | 14003 (46.4) | 12677 (46.9) |
| Sex |  |  |  |  |
| Male | 51254 (60.5) | 33506 (56.2) | 15792 (52.3) | 14048 (51.9) |
| Female | 33485 (39.5) | 26126 (43.8) | 14385 (47.7) | 13007 (48.1) |
| State of residence |  |  |  |  |
| Iowa | 53647 (63.3) | 40600 (68.1) | 22512 (74.6) | 20456 (75.6) |
| North Carolina | 31092 (36.7) | 19032 (31.9) | 7665 (25.4) | 6599 (24.4) |
| Race |  |  |  |  |
| non-White | 2070 (2.5) | 1079 (1.8) | 320 (1.1) | 279 (1.0) |
| White | 80819 (97.5) | 58499 (98.2) | 29837 (98.9) | 26758 (99.0) |
| Missing | 1850 | 54 | 20 | 18 |
| Hispanic ethnicity |  |  |  |  |
| No | 79099 (99.0) | 57113 (99.0) | 29184 (99.2) | 26163 (99.2) |
| Yes | 796 (1.0) | 555 (1.0) | 238 (0.8) | 214 (0.8) |
| Missing | 4844 | 1964 | 755 | 678 |
| Educational attainment |  |  |  |  |
| ≤ High school diploma/GED | 42202 (52.0) | 30133 (50.5) | 14462 (47.9) | 13025 (48.1) |
| Some college/2-year degree | 23632 (29.1) | 17891 (30.0) | 9284 (30.8) | 8328 (30.8) |
| College graduate | 15265 (18.8) | 11608 (19.5) | 6431 (21.3) | 5702 (21.1) |
| Missing | 3640 | 0 | 0 | 0 |
| Smoking status |  |  |  |  |
| Never smoked | 49912 (60.3) | 36788 (61.7) | 19210 (63.7) | 17245 (63.7) |
| Former smoker | 21481 (25.9) | 15323 (25.7) | 7839 (26.0) | 7008 (25.9) |
| Currrent smoker | 11442 (13.8) | 7521 (12.6) | 3128 (10.4) | 2802 (10.4) |
| Missing | 1904 | 0 | 0 | 0 |
| Reported pre-enrollment diabetes diagnosis |  |  |  |  |
| No | 76435 (96.8) | 55777 (96.9) | 28516 (97.1) | 25562 (97.0) |
| Yes | 2536 (3.2) | 1784 (3.1) | 855 (2.9) | 784 (3.0) |
| Missing | 5768 | 2071 | 806 | 709 |
| Reported pre-enrollment kidney disease (excluding kidney stones) diagnosis |  |  |  |  |
| No | 77809 (98.2) | 56681 (98.3) | 28897 (98.2) | 25905 (98.2) |
| Yes | 1390 (1.8) | 989 (1.7) | 534 (1.8) | 489 (1.9) |
| Missing | 5540 | 1962 | 746 | 661 |
| Weight classification |  |  |  |  |
| Underweight or normal (BMI < 25) | 24075 (35.7) | 19265 (35.5) | 10881 (36.4) | 9671 (36.1) |
| Overweight (25 ≤ BMI < 30) | 28956 (42.9) | 23312 (43.0) | 12738 (42.6) | 11467 (42.8) |
| Obese (30 ≤ BMI < 35) | 14425 (21.4) | 11644 (21.5) | 6253 (20.9) | 5654 (21.1) |
| Missing | 17283 | 5411 | 305 | 263 |
| Lifetime days any pesticide use |  |  |  |  |
| Never | 14257 (19.0) | 11150 (20.5) | 5584 (20.4) | 5026 (20.5) |
| Low (3-64 days) | 20777 (27.6) | 14760 (27.1) | 7588 (27.7) | 6738 (27.5) |
| Medium (88-245 days) | 21091 (28.1) | 15022 (27.6) | 7714 (28.2) | 6914 (28.2) |
| High (368-7000 days) | 19067 (25.4) | 13462 (24.8) | 6481 (23.7) | 5863 (23.9) |
| Missing | 9547 | 5238 | 2810 | 2514 |
| Duration of residence at the enrollment address |  |  |  |  |
| < 10 | 17311 (28.3) | 14229 (27.9) | 6826 (25.8) | 6063 (25.1) |
| 10-19 | 16435 (26.9) | 13908 (27.3) | 6975 (26.3) | 6364 (26.3) |
| 20-29 | 13810 (22.6) | 11620 (22.8) | 6349 (24.0) | 5863 (24.3) |
| ≥ 30 | 13639 (22.3) | 11220 (22.0) | 6353 (24.0) | 5885 (24.3) |
| Missing | 23544 | 8655 | 3674 | 2880 |
|  |  |  |  |  |
|  |  |  |  |  |
| Abbreviations: AHS, *Agricultural Health Study*; GED, General Equivalency Diploma; BMI, body mass index | | | | |
| ^a^Private applicators and their spouses enrolled in the study. Enrollment occurred in 1993-1997. | | | | |
| ^b^Analytical sample for water nitrate analysis. Eligible participants had complete water nitrate estimates and did not have end-stage renal disease before study enrollment. | | | | |
| ^c^Analytical sample for dietary nitrate/nitrite analysis. Eligible participants had complete dietary nitrate and nitrite intake estimates ascertained from a diet history questionnaire administered (1999-2003) and did not have end-stage renal disease before administration of the diet questionnaire. | | | | |
| ^d^Analytical sample for a sub-analysis examining effect measure modification of the association between water nitrate exposure and end-stage renal disease by heme iron and vitamin C intake. Participants in this analysis had complete water nitrate and dietary data and did not have end-stage renal disease before study enrollment. | | | | |

| Table S5. Sensitivity analyses of the association between average water nitrate exposure and risk of end-stage renal disease among AHS participants, 1993-2018 | | | | |
| --- | --- | --- | --- | --- |
| **Sensitivity Analysis** | **Average NO_3_-N (mg/L)** | **Total Cases** | **Total N** | **HR (95% CI)^a^** |
|  |  |  |  |  |
| Exclude pre-enrollment diabetes | T1 (0-1) | 109 | 19332 | Referent |
|  | T2 (1-2.4) | 134 | 19196 | 1.00 (0.77, 1.30) |
|  | T3 (2.4-46) | 92 | 19320 | 0.84 (0.64, 1.11) |
|  |  |  |  |  |
| Additionally adjust for body mass index | T1 (0-1) | 128 | 17842 | Referent |
|  | T2 (1-2.4) | 174 | 18216 | 1.09 (0.87, 1.38) |
|  | T3 (2.4-46) | 121 | 18163 | 0.92 (0.72, 1.18) |
|  |  |  |  |  |
| Additionally adjust for lifetime days use of pesticides | T1 (0-1) | 139 | 18180 | Referent |
|  | T2 (1-2.4) | 168 | 18091 | 0.98 (0.78, 1.23) |
|  | T3 (2.4-46) | 124 | 18123 | 0.89 (0.70, 1.13) |
|  |  |  |  |  |
| Additionally adjust for year of study enrollment | T1 (0-1) | 148 | 19858 | Referent |
|  | T2 (1-2.4) | 186 | 19856 | 1.02 (0.82, 1.27) |
|  | T3 (2.4-46) | 135 | 19918 | 0.91 (0.72, 1.14) |
|  |  |  |  |  |
| Restrict to participants who lived on their address at enrollment for ≥10 years | T1 (0-1) | 90 | 11158 | Referent |
|  | T2 (1-2.4) | 141 | 12754 | 1.13 (0.86, 1.48) |
|  | T3 (2.4-43) | 103 | 12836 | 1.02 (0.77, 1.35) |
|  |  |  |  |  |
| Assess alternative exposure-response trends using quartile exposure | Q1 (0-0.8) | 110 | 14909 | Referent |
|  | Q2 (0.8-1.5) | 132 | 14907 | 1.14 (0.89, 1.47) |
|  | Q3 (1.5-3.1) | 133 | 14908 | 0.93 (0.72, 1.20) |
|  | Q4 (3.1-46) | 94 | 14908 | 0.96 (0.72, 1.27) |
|  |  |  |  |  |
| Examine effect of exposure to extreme levels of water nitrate (above 90th percentile) | T1 (0-1) | 148 | 19858 | Referent |
|  | T2 (1-2.4) | 186 | 19856 | 1.02 (0.82, 1.27) |
|  | T3-P90 (2.4-7.3) | 101 | 13959 | 0.91 (0.71, 1.17) |
|  | >P90 (7.3-46) | 34 | 5959 | 0.89 (0.61, 1.31) |
|  |  |  |  |  |
|  |  |  |  |  |
| Abbreviations: AHS, Agricultural Health Study; HR, hazard ratio; CI, confidence interval; P90, 90th percentile | | | | |
| ^a^Cox proportional hazards models accounted for age, sex, state of residence, education, and smoking status. Other covariates were additionally adjusted in specific models (see labels in the first column). | | | | |

| Table S6. Associations between dietary nitrate and nitrite exposure and risk of end-stage renal disease among male pesticide applicators and among female spouses in the AHS, 1999-2018 | | | | | | | | | | | |
| --- | --- | --- | --- | --- | --- | --- | --- | --- | --- | --- | --- |
| **Exposure** | **Male pesticide applicators** | | | |  | **Female spouses** | | | |  | **Interaction p** |
|  | **Tertile** | **Total Cases (n=129)** | **Total N (n=15,737)** | **HR (95% CI)^a^** |  | **Tertile** | **Total Cases (n=76)** | **Total N (n=13,948)** | **HR (95% CI)^a^** |  |  |
| Nitrate, diet total (mg NO_3_/day) | T1 (4.6-45.1) | 41 | 5143 | Referent |  | T1 (4.6-45.1) | 21 | 4775 | Referent |  | 0.85 |
|  | T2 (45.1-74.5) | 33 | 5344 | 0.69 (0.43, 1.11) |  | T2 (45.1-74.5) | 21 | 4544 | 0.80 (0.43, 1.48) |  |  |
|  | T3 (74.5-539.2) | 55 | 5250 | 1.01 (0.63, 1.63) |  | T3 (74.5-553.6) | 34 | 4629 | 1.05 (0.56, 1.97) |  |  |
|  |  |  |  |  |  |  |  |  |  |  |  |
| Nitrite, diet total (mg NO_2_/day) | T1 (0.1-0.9) | 29 | 3559 | Referent |  | T1 (0.1-0.9) | 34 | 6316 | Referent |  | 0.79 |
|  | T2 (0.9-1.3) | 34 | 5105 | 0.91 (0.54, 1.53) |  | T2 (0.9-1.3) | 20 | 4834 | 0.66 (0.36, 1.22) |  |  |
|  | T3 (1.4-4.6) | 66 | 7073 | 1.44 (0.78, 2.66) |  | T3 (1.4-3.8) | 22 | 2798 | 0.98 (0.42, 2.29) |  |  |
|  |  |  |  |  |  |  |  |  |  |  |  |
|  |  |  |  |  |  |  |  |  |  |  |  |
| Abbreviations: AHS, Agricultural Health Study; HR, hazard ratio; CI, confidence interval | | | | | | | | | | | |
| ^a^Cox proportional hazards models accounted for age, state of residence, education, smoking status, and total caloric intake. | | | | | | | | | | | |

| Table S7. Associations between dietary nitrate and nitrite exposure and risk of end-stage renal disease among AHS participants, stratified by state of residence, 1999-2018 | | | | | | | | | | | |
| --- | --- | --- | --- | --- | --- | --- | --- | --- | --- | --- | --- |
| **Exposure** | **Iowa Residence** | | | |  | **North Carolina Residence** | | | |  | **Interaction p** |
|  | **Tertile** | **Total Cases (n=122)** | **Total N (n=22,512)** | **HR (95% CI)^a^** |  | **Tertile** | **Total Cases (n=84)** | **Total N (n=7,665)** | **HR (95% CI)^a^** |  |  |
| Nitrate, diet total (mg NO_3_/day) | T1 (4.6-45.1) | 40 | 7957 | Referent |  | T1 (4.6-45.1) | 22 | 2095 | Referent |  | 0.65 |
|  | T2 (45.1-74.5) | 36 | 7623 | 0.81 (0.51, 1.29) |  | T2 (45.1-74.5) | 18 | 2424 | 0.60 (0.32, 1.14) |  |  |
|  | T3 (74.5-553.6) | 46 | 6932 | 1.04 (0.64, 1.70) |  | T3 (74.5-539.2) | 44 | 3146 | 1.01 (0.55, 1.83) |  |  |
|  |  |  |  |  |  |  |  |  |  |  |  |
| Nitrite, diet total (mg NO_2_/day) | T1 (0.1-0.9) | 46 | 7597 | Referent |  | T1 (0.1-0.9) | 17 | 2454 | Referent |  | 0.02 |
|  | T2 (0.9-1.3) | 36 | 7711 | 0.70 (0.43, 1.13) |  | T2 (0.9-1.3) | 18 | 2411 | 1.16 (0.58, 2.33) |  |  |
|  | T3 (1.4-4.6) | 40 | 7204 | 0.77 (0.40, 1.50) |  | T3 (1.4-4.3) | 49 | 2800 | 2.94 (1.38, 6.28) |  |  |
|  |  |  |  |  |  |  |  |  |  |  |  |
|  |  |  |  |  |  |  |  |  |  |  |  |
| Abbreviations: AHS, Agricultural Health Study; HR, hazard ratio; CI, confidence interval | | | | | | | | | | | |
| ^a^Cox proportional hazards models accounted for age, sex, education, smoking status, and total caloric intake. | | | | | | | | | | | |

| Table S8. Sensitivity analyses of the associations between dietary nitrate and nitrite exposure and risk of end-stage renal disease among AHS participants, 1999-2018 | | | | | |
| --- | --- | --- | --- | --- | --- |
| **Sensitivity Analysis** | **Exposure** | | **Total Cases** | **Total N** | **HR (95% CI)^a^** |
| Exclude pre-enrollment diabetes | Nitrate, diet total (mg NO_3_/day) | T1 (4.6-45.1) | 51 | 9834 | Referent |
|  |  | T2 (45.1-74.5) | 41 | 9754 | 0.68 (0.44, 1.03) |
|  |  | T3 (74.5-553.6) | 63 | 9734 | 0.88 (0.57, 1.36) |
|  |  |  |  |  |  |
|  | Nitrite, diet total (mg NO_2_/day) | T1 (0.1-0.9) | 46 | 9769 | Referent |
|  |  | T2 (0.9-1.3) | 46 | 9826 | 0.94 (0.61, 1.47) |
|  |  | T3 (1.4-4.6) | 63 | 9727 | 1.21 (0.68, 2.14) |
|  |  |  |  |  |  |
| Additionally adjust for body mass index | Nitrate, diet total (mg NO_3_/day) | T1 (4.6-45.1) | 60 | 9958 | Referent |
|  |  | T2 (45.1-74.5) | 54 | 9940 | 0.75 (0.51, 1.09) |
|  |  | T3 (74.5-553.6) | 90 | 9974 | 1.05 (0.72, 1.54) |
|  |  |  |  |  |  |
|  | Nitrite, diet total (mg NO_2_/day) | T1 (0.1-0.9) | 61 | 9923 | Referent |
|  |  | T2 (0.9-1.3) | 54 | 10013 | 0.87 (0.58, 1.29) |
|  |  | T3 (1.4-4.6) | 89 | 9936 | 1.42 (0.87, 2.31) |
|  |  |  |  |  |  |
| Additionally adjust for lifetime days use of pesticides | Nitrate, diet total (mg NO_3_/day) | T1 (4.6-45.1) | 55 | 9098 | Referent |
|  |  | T2 (45.1-74.5) | 48 | 9119 | 0.71 (0.47, 1.06) |
|  |  | T3 (74.5-553.6) | 83 | 9150 | 1.02 (0.69, 1.52) |
|  |  |  |  |  |  |
|  | Nitrite, diet total (mg NO_2_/day) | T1 (0.1-0.9) | 52 | 8864 | Referent |
|  |  | T2 (0.9-1.3) | 49 | 9226 | 0.90 (0.59, 1.37) |
|  |  | T3 (1.4-4.6) | 85 | 9277 | 1.51 (0.90, 2.52) |
|  |  |  |  |  |  |
| Additionally adjust for year of study enrollment | Nitrate, diet total (mg NO_3_/day) | T1 (4.6-45.1) | 62 | 10052 | Referent |
|  |  | T2 (45.1-74.5) | 54 | 10047 | 0.73 (0.50, 1.06) |
|  |  | T3 (74.5-553.6) | 90 | 10078 | 1.04 (0.71, 1.52) |
|  |  |  |  |  |  |
|  | Nitrite, diet total (mg NO_2_/day) | T1 (0.1-0.9) | 63 | 10051 | Referent |
|  |  | T2 (0.9-1.3) | 54 | 10122 | 0.84 (0.57, 1.24) |
|  |  | T3 (1.4-4.6) | 89 | 10004 | 1.35 (0.83, 2.20) |
|  |  |  |  |  |  |
| Examine effect of exposure to extreme levels of dietary intake (above 90th percentile) | Nitrate, diet total (mg NO_3_/day) | T1 (4.6-45.1) | 62 | 10052 | Referent |
|  |  | T2 (45.1-74.5) | 54 | 10047 | 0.73 (0.50, 1.06) |
|  |  | T3-P90 (74.5-125.8) | 59 | 7061 | 1.02 (0.69, 1.51) |
|  |  | >P90 (125.8-553.6) | 31 | 3017 | 1.12 (0.68, 1.85) |
|  |  |  |  |  |  |
|  | Nitrite, diet total (mg NO_2_/day) | T1 (0.1-0.9) | 63 | 10051 | Referent |
|  |  | T2 (0.9-1.3) | 54 | 10122 | 0.92 (0.62, 1.37) |
|  |  | T3-P90 (1.4-1.9) | 54 | 7048 | 1.42 (0.87, 2.33) |
|  |  | >P90 (1.9-4.6) | 35 | 2956 | 2.47 (1.20, 5.09) |
|  |  |  |  |  |  |
|  |  |  |  |  |  |
| Abbreviations: AHS, Agricultural Health Study; HR, hazard ratio; CI, confidence interval | | | | | |
| ^a^Cox proportional hazards models accounted for age, sex, state of residence, education, smoking status, and total caloric intake. Other covariates were additionally adjusted in specific models (see labels in the first column). | | | | | |

| Table S9. Associations between total dietary nitrate and nitrite exposures (expressed as nutrient densities, i.e., per 1,000 kcal) and risk of end-stage renal disease among AHS participants (N=30,177), 1999-2018 | | | | |
| --- | --- | --- | --- | --- |
| **Exposure** |  | **Total Cases (n=206)** | **Total N (n=30,177)** | **HR (95% CI)^a^** |
| Nitrate, diet total (mg NO_3_/day/1,000 kcal) | T1 (5.5-25.3) | 62 | 10052 | Referent |
|  | T2 (25.3-40.3) | 54 | 10047 | 1.21 (0.84, 1.72) |
|  | T3 (40.3-525) | 90 | 10078 | 1.05 (0.72, 1.53) |
|  |  |  |  |  |
| Nitrite, diet total (mg NO_2_/day/1,000 kcal) | T1 (0.1-0.6) | 63 | 10051 | Referent |
|  | T2 (0.6-0.7) | 54 | 10122 | 1.23 (0.85, 1.77) |
|  | T3 (0.7-2.2) | 89 | 10004 | 1.29 (0.90, 1.85) |
|  |  |  |  |  |
|  |  |  |  |  |
| Abbreviations: AHS, Agricultural Health Study; HR, hazard ratio; CI, confidence interval | | | | |
| ^a^Cox proportional hazards models accounted for age, sex, state of residence, education, and smoking status. | | | | |

Dietary nitrate/nitrite analysis

Completed the DHQ

(N=33,254)

Did not have ESRD before completion of DHQ

(N=33,210)

Complete dietary exposure estimates

(N=31,130)

Complete covariate information

(N=30,177)

Water nitrate analysis

Enrolled cohort (private pesticide applicators and their spouses, commercial applicators)

(N=89,655)

Private applicators and their spouses

(N=84,739)

Did not have ESRD before study enrollment

(N=84,667)

Primary drinking water source was not missing

(N=72,173)

Primary drinking water source was a private well or public water supply

(N=69,148)

Complete water nitrate exposure estimates

(N=62,291)

Complete covariate information

(N=59,632)

Figure S1. Participant eligibility for the analyses of water nitrate and dietary nitrate/nitrite in relation to ESRD hazards in the Agricultural Health Study

Abbreviations: ESRD, end-stage renal disease; DHQ, dietary history questionnaire
